# Childhood maltreatment and aggressive behavior among college students: a serial mediation model of authenticity and alexithymia

**DOI:** 10.3389/fpsyt.2024.1478127

**Published:** 2024-10-11

**Authors:** Jingya Zhou, Meiting Wei, Qing Xie

**Affiliations:** ^1^ Department of Public Order Administration, Hunan Police Academy, Changsha, China; ^2^ Faculty of Education, Yunnan Normal University, Kunming, China; ^3^ Department of Management, Hunan Police Academy, Changsha, China

**Keywords:** childhood maltreatment, aggressive behavior, authenticity, alexithymia, college students

## Abstract

**Introduction:**

Aggressive behavior among college students is a concerning issue that not only affects the mental health and personal development of those involved but also poses a threat to societal stability. Existing literature has consistently demonstrated a positive correlation between childhood maltreatment and aggressive behavior. However, the specific mechanisms through which childhood maltreatment leads to aggressive behavior remain unclear. This study aims to explore the impact of childhood maltreatment on aggressive behavior among college students and to examine the mediating roles of authenticity and alexithymia in this relationship.

**Methods:**

To investigate these relationships, we conducted an online survey among 1,148 Chinese college students. Participants completed the Childhood Trauma Questionnaire-Short Form (CTQ-SF), Authenticity Scale, Toronto Alexithymia Scale (TAS-20), and 12-item Aggression Questionnaire (12-AQ). These instruments allowed us to measure the variables of interest and to analyze the potential mediating effects of authenticity and alexithymia.

**Results:**

The findings of our study indicate that both authenticity and alexithymia mediate the positive relationship between childhood maltreatment and aggressive behavior. Specifically, the mediating effect of authenticity was 0.04 (95% CI [0.01, 0.06]), while that of alexithymia was 0.10 (95% CI [0.07, 0.13]). Moreover, we observed a chain-mediating effect involving both authenticity and alexithymia, with a chain-mediating effect of 0.03 (95% CI [0.02, 0.05]).

**Conclusions:**

This study demonstrates that childhood maltreatment can positively predict aggressive behavior in college students, and this relationship is mediated individually and sequentially by authenticity and alexithymia. Our findings contribute valuable insights to the existing research on aggressive behavior and provide a theoretical framework for developing interventions aimed at reducing aggressive behaviors among college students.

## 1 Introduction

Aggressive behavior, also known as offensive behavior, is a goal directed motivational behavior that intentionally causes physical injury or psychological pain to others, rather than doing so accidentally or unconsciously (1). With the frequent occurrence of a series of incidents including criminal behavior, campus violence, and dating violence among college students (2–4), aggressive behavior in this group has become a focal issue in academic research. A survey showed that approximately one-third of Chinese college students exhibit a tendency towards moderate or higher levels of aggressive behavior (5). The World Health Organization’s report shows that approximately 37% of global violent crimes are committed by individuals aged 15-29, including the college student population (6). Meanwhile, aggressive behavior poses significant risks, not only to the victims’ physical and mental health (7, 8), but also to the aggressors themselves, impairing their personality development (9, 10) and contributing to higher crime rates (11, 12). Therefore, it is necessary to explore the influencing factors and mechanisms behind aggressive behavior among college students.

Childhood maltreatment refers to actions by individuals who have a duty to care for and supervise a child, which are sufficient to cause actual or potential harm to the child’s health, survival, growth, development and dignity (13). In China, the overall prevalence of college students having experienced one or more forms of childhood maltreatment—including sexual abuse, emotional abuse, physical abuse, emotional neglect, and physical neglect—is 64.7% (14). In line with Ecological Systems Theory, childhood maltreatment, functioning as a microsystem, will exert an immediate negative influence on an individual’s development, leading to aggressive behavior among college students (15, 16). Previous research confirms that college students who have endured childhood maltreatment often exhibit lower levels of self-esteem, well-being, and security, as well as higher levels of anxiety and depression, which are risk factors for aggression (17–20). Other empirical evidence directly demonstrates that childhood maltreatment is a significant risk factor for aggressive behavior among college students (21, 22). Furthermore, Attachment Theory suggests that the relational patterns between a child and their caregivers are gradually internalized as schemas of self-other relationships, influencing an individual’s attitudes and behaviors towards others (23). College students who have experienced childhood maltreatment are more likely to develop insecure styles of adult attachment, manifesting as attachment anxiety and attachment avoidance (24). Meanwhile, previous research has demonstrated that these negative relational patterns more readily lead to anger and hostility toward others, thereby increasing aggressive behavior (25, 26). Therefore, this study posits that childhood maltreatment positively predicts aggressive behavior in college students.

Authenticity is often defined as the sense of being true to one’s real self (27). According to Self-Determination Theory, individuals are considered authentic when their behaviors are autonomous and reflect their true selves (28, 29). Childhood abuse experiences can decrease an individual’s sense of authenticity. This occurs because individuals who experience neglect or abuse from their parents are more likely to ignore or suppress their true thoughts and emotions, thereby developing a pattern of inauthentic self-expression (30, 31). Consistently, Li and Bi found that excessive control by parents can diminish the authenticity of Chinese college students (32). Theran and Han demonstrated that emotional maltreatment by parents could positively predict inauthenticity in female college students (33). Meanwhile, based on the Meaning Maintenance Model, low authenticity can diminish an individual’s sense of meaning (34). When individuals interact with others, they are more inclined to restore their sense of meaning through aggressive behaviors (35). Some evidence indicates that a threat to the sense of meaning can foster hostility and aggressive behavior (36, 37). Importantly, a cross-sectional study conducted by Pinto and associates revealed that individuals with lower levels of authentic living are more likely to exhibit aggressive responses (38). In conjunction with Self-Determination Theory, this suggests that childhood abuse can reduce an individual’s sense of meaning by diminishing their authenticity, thereby increasing the likelihood of the individual engaging in aggressive behavior.

Alexithymia is a relatively stable personality construct, primarily characterized by difficulties in identifying feelings, difficulties in describing feelings, and an externally oriented style of thinking (39, 40). Therefore, alexithymia may mediate the relationship between childhood maltreatment and aggressive behavior in college students (41). On one hand, childhood abuse may hinder the growth and development of brain structures related to emotions, thereby affecting the development of emotional processes (42). Studies have validated that individuals who endured repeated physical or verbal mistreatment by their parents during childhood often display anomalies in the hippocampus and prefrontal cortex (43–45). Additionally, based on the Self-defense Mechanism Hypothesis, the experience of abuse and neglect is often intolerable for children, leading them to be more inclined to adopt emotional numbing or dissociation as a defensive mechanism for self-protection (46). This evidence suggests that childhood abuse is a risk factor for the development of alexithymia. On the other hand, alexithymia impedes an individual’s capacity for empathy towards others and the recognition of one’s own emotions, thus blocking the channels for expressing and dissipating anxiety or anger (47, 48). When individuals encounter interpersonal conflicts, they may find it difficult to adopt effective stress-coping strategies to alleviate the conflict, which increases the likelihood of aggressive behavior. As a result, those with pronounced alexithymia are at a higher risk of exhibiting aggressive actions. Ample evidence indicates that higher levels of alexithymia are associated with increased aggressive behavior (49, 50), a finding supported by neurobiological evidence (51).

Self-alienation is an important dimension of authenticity, which refers to a subjective experience of not knowing who one is and being disconnected from one’s core self (52). This suggests that individuals with lower levels of authenticity often find it difficult to discern their true thoughts and express their true feelings, which can lead to increased alexithymia (53). Meanwhile, the General Aggression Model (GAM) suggests that distal environmental factors can influence aggressive behavior by altering an individual’s personality traits (54). According to the GAM, childhood maltreatment, authenticity, alexithymia, and aggressive behavior in college students may form a comprehensive process leading to aggression. In other words, childhood maltreatment, as a distal environmental factor, hampers individuals from authentically expressing themselves and living genuinely, exacerbating difficulties in recognizing and expressing emotions and leading to the development of alexithymia (30, 46). Alexithymia, in turn, hinders individuals from employing appropriate coping strategies to resolve conflicts and regulate negative emotions, thereby leading to aggressive behavior (47). Based on this, the present study posits that childhood maltreatment may influence aggressive behavior through a chain mediation effect involving authenticity and alexithymia.

Previous research has found varying degrees of correlation between childhood maltreatment, authenticity, alexithymia, and aggressive behavior. Additionally, authenticity and alexithymia are both considered mediating factors in the relationship between childhood maltreatment and aggressive behavior, with low authenticity potentially exacerbating alexithymia. Based on these studies, authenticity and alexithymia may not only independently mediate the relationship between childhood maltreatment and aggressive behavior in college students but may also exhibit a chain-mediated effect. This study aims to explore this psychological mechanism through a cross-sectional questionnaire survey (*N* = 1,148) among Chinese college students.

## 2 Materials and methods

### 2.1 Participants

Considering that Chinese universities organize and manage students in classes (approximately 50 students per class), we employed a cluster sampling method, using university class as the sampling unit. A total of 1,200 students were recruited from four universities in Hunan Province, China. Participants received the survey link on the *Credamo* platform after providing informed consent. They voluntarily initiated the questionnaire after reading the introduction on the homepage and had the option to withdraw from the study at any time. After excluding 52 invalid responses (failed the attention check or provided patterned responses), the final sample size consisted of 1,148 participants. The basic information of the sample is presented in **Table 1**. Upon submitting the questionnaire, all participants were compensated with 3 RMB (approximately 0.42 USD).

**Table 1:** Distribution of sample characteristic.

Variables	Groups	N	Proportion
Gender	Male	540	47.0%
	Female	608	53.0%
Age	18	368	32.1%
	19	418	36.4%
	20	244	21.3%
	21	84	7.3%
	22	26	2.3%
	23	8	0.7%
Hometown	Urban areas	466	40.6%
	Rural areas	682	59.4%

*N* = 1,148.

The study complied with the Declaration of Helsinki and approved by the Institutional Review Board of corresponding author’s institution. Before commencing the research, we reached out to the administrative authorities of the schools involved and secured authorization for the questionnaire survey, as well as obtained informed consent from the student participants. The participants are all over the age of 18, therefore they are capable of making independent decisions without the consent of a guardian. All participants voluntarily participated in the survey after fully understanding the purpose, methods, potential risks and benefits of the study.

### 2.2 Measures

#### 2.2.1 Childhood maltreatment

Childhood maltreatment was measured using the Childhood Trauma Questionnaire-Short Form (CTQ-SF) developed by Bernstein et al. (55), which was revised into Chinese version by Zhao et al. (56). The scale is widely used and has good reliability and validity (56–58). The questionnaire comprises 28 items, of which 25 are clinical items, including five subscales: sexual abuse, emotional abuse, physical abuse, emotional neglect, and physical neglect. All items were rated on a scale of 1 to 5 (1 = never to 5 = always), with higher scores indicating greater childhood maltreatment. In this study, the Cronbach’s alpha coefficient for this questionnaire was 0.90, and the Cronbach’s alpha coefficients for the five subscales were 0.90, 0.78, 0.86, 0.85 and 0.75, respectively, indicating good reliability.

#### 2.2.2 Authenticity

Authenticity was measured using the Authenticity Scale developed by Wood et al. (59), which was revised into Chinese version by Song et al. (60). The scale is widely used for measuring individual authenticity and has good reliability and validity (32, 60, 61). The scale consists of 12 items, which are divided into three subscales: authentic living, self-alienation and accepting external influence. All items were rated on a scale of 1 to 7 (1 = strongly disagree to 7 = strongly agree), with higher scores indicating greater authenticity. In this study, the Cronbach’s alpha coefficient for this scale was 0.81, and the Cronbach’s alpha coefficients for the three subscales were 0.76, 0.76 and 0.85, respectively, indicating good reliability.

#### 2.2.3 Alexithymia

Alexithymia was measured using The Toronto Alexithymia Scale (TAS-20) developed by Taylor et al. (62), which was revised into Chinese version by Yuan et al. (63). The scale has been used by many researchers to measure individual alexithymia and has good reliability and validity (12, 63–65). The scale comprises 20 items, which are organized into three subscales: difficulties in identifying feelings, difficulties in describing feelings, and external-oriented thinking. All items were rated on a scale of 1 to 5 (1 = strongly disagree to 5 = strongly agree), with higher scores indicating greater alexithymia. In our study, the Cronbach’s alpha coefficient for this scale was 0.88, and the Cronbach’s alpha coefficients for the three subscales were 0.76, 0.80 and 0.78, respectively, indicating good reliability.

#### 2.2.4 Aggressive behavior

Aggressive Behavior was measured using the 12-item Aggression Questionnaire (12-AQ) developed by Bryant and Smith (66), which was revised into Chinese version by Zhang et al. (67). The scale is widely used for measuring individual level of aggression and has good reliability and validity (67–69). This questionnaire comprises four subscales: physical attacks, verbal attacks, anger and hostility. All items were rated on a scale of 1 to 5 (1 = strongly disagree to 5 = strongly agree), with higher scores indicating more aggressive behavior. In this study, the Cronbach’s alpha coefficient for this scale was 0.88, indicating good internal consistency. The Cronbach’s alpha coefficients for the four subscales were 0.74, 0.69, 0.68 and 0.76, respectively, indicating good reliability.

### 2.3 Data analysis

Descriptive statistics, correlation analysis, and mediation effect tests were conducted using SPSS 23.0. Initially, we conducted a common method bias test. Subsequently, we compiled descriptive statistics and calculated the correlation coefficients for the key variables. Finally, we employed Model 6 from the PROCESS macro within SPSS 23.0 to assess serial mediation and the significance of the serial mediation effect was tested using the Bootstrap method (70).

## 3 Results

### 3.1 Common method bias test

The Harman single-factor test was employed to examine common method bias. The results indicated that 13 factors had eigenvalues greater than 1, and the variance explained by the first unrotated factor was 21.89% (less than the critical threshold of 40%). Thus, common method bias in our study was not serious.

### 3.2 Descriptive statistics and correlation analysis

The results of the descriptive statistics and correlation analyses for our focal variables were presented in **Table 2**. Childhood maltreatment was negatively correlated with authenticity (*r* = -0.30, *p* < 0.001), and was positively correlated with alexithymia (*r* = 0.42, *p* < 0.001) and aggressive behavior (*r* = 0.36, *p* < 0.001). Authenticity was negatively correlated with alexithymia (*r* = -0.45, *p* < 0.001) and aggressive behavior (*r* = -0.32, *p* < 0.001). Furthermore, alexithymia was positively correlated with aggressive behavior (*r* = 0.45, *p* < 0.001). These correlations among the variables are consistent with our hypotheses.

**Table 2:** Descriptive statistics and correlations among focus variables.

Variables	M	SD	1	2	3	4
1. Childhood Maltreatment	1.62	0.40	1.00			
2. Authenticity	4.66	0.85	-0.30^***^	1.00		
3 .Alexithymia	2.67	0.54	0.42^***^	-0.45^***^	1.00	
4. Aggressive Behavior	2.47	0.76	0.36^***^	-0.32^***^	0.45^***^	1.00

*N* = 1,148. ***p < 0.001 (two-tailed).

### 3.3 Mediating analysis

**Figure 1:**
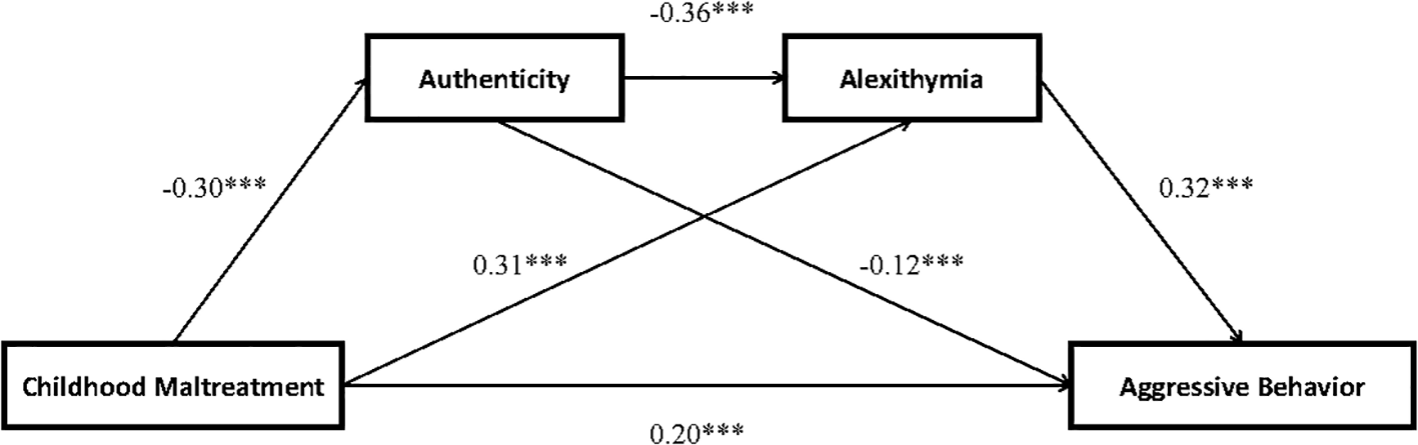
Standardized path coefficients of research model. *N* = 1,148. ***p < 0.001 (two-tailed).

Model 6 of the PROCESS macro was conducted to test the mediation effects of authenticity and alexithymia. The standardized coefficients for each pathway are shown in **Figure 1**. After controlling for gender and age, the total effect of childhood maltreatment on aggressive behavior was significant (*β* = 0.37, *t* = 13.25, *p* < 0.001). This indicates that childhood maltreatment can positively predict aggressive behavior.

Furthermore, as shown in **Tables 3** and **4**, childhood maltreatment was negatively related to authenticity (*β* = -0.30, *t* = -10.86, *p* < 0.001), and authenticity was negatively related to aggressive behavior (*β* = -0.12, *t* = -4.17, *p* < 0.001). The bias-corrected non-parametric bootstrap method indicated that the indirect effect of authenticity was significant (*Estimate* = 0.04, *SE* = 0.01, 95%CI [0.01, 0.06]). This indicates that authenticity plays a mediating role in the relationship between childhood maltreatment and aggressive behavior.

**Table 3:** The analytic results of serial mediation model.

Independent variables	Dependent variables					
	Authenticity		Alexithymia		Aggressive Behavior	
	*β*	*t*	*β*	*t*	*β*	*t*
Childhood maltreatment	-0.30	-10.86^***^	0.31	12.00^***^	0.20	6.81^***^
Authenticity			-0.36	-13.57^***^	-0.12	-4.17^***^
Alexithymia					0.32	10.47^***^
Gender	-0.17	-2.99^**^	-0.14	-2.71^**^	0.07	1.80
Age	0.06	2.18^*^	-0.05	-2.09^*^	0.05	1.36
*R^2^*	0.11		0.30		0.25	
*F*	43.32^***^		120.13^***^		78.12^***^	

*N* = 1,148. *p < 0.05, **p < 0.01, ***p < 0.001 (two-tailed). All variables in the model are standardized before being incorporated into the regression equation.

**Table 4:** The mediating role of authenticity and alexithymia.

	Estimate (SE)	95% CI	Proportion
Indirect effect (Childhood Maltreatment−Authenticity−Aggressive Behavior)	0.04 (0.01)	[0.01, 0.06]	10.81%
Indirect effect (Childhood Maltreatment−Alexithymia−Aggressive Behavior)	0.10 (0.01)	[0.07, 0.13]	27.03%
Indirect effect (Childhood Maltreatment−Authenticity−Alexithymia−Aggressive Behavior)	0.03 (0.01)	[0.02, 0.05]	8.11%
Total indirect effect	0.17(0.02)	[0.13, 0.21]	45.95%
Direct effect	0.20 (0.03)	[0.14, 0.25]	54.05%
Total effect	0.37 (0.03)	[0.31, 0.42]	

Childhood maltreatment was positively related to alexithymia (*β* = 0.31, *t* = 12.00, *p* < 0.001), and alexithymia was positively related to aggressive behavior (*β* = 0.32, *t* = 10.47, *p* < 0.001). The bias-corrected non-parametric bootstrap method indicated that the indirect effect of alexithymia was significant (*Estimate* = 0.10, *SE* = 0.01, 95%CI [0.07, 0.13]). This indicates that alexithymia plays a mediating role in the relationship between childhood maltreatment and aggressive behavior.

Importantly, authenticity was negatively related to alexithymia (*β* = -0.36, *t* = -13.57, *p* < 0.001). The bias-corrected non-parametric bootstrap method revealed that the serial indirect effect was significant (*Estimate* = 0.03, *SE* = 0.01, 95%CI [0.02, 0.05]). This indicates that authenticity and alexithymia play a chain mediation role in the relationship between childhood maltreatment and aggressive behavior.

Meanwhile, the direct effect of childhood maltreatment on aggressive behavior remained significant (*β* = 0.20, *t* = 6.80, *p* < 0.001).

Additionally, we employed the Monte Carlo Power Analysis for Indirect Effects (https://schoemanna.shinyapps.io/mc_power_med/) to examine the statistical power of the chain mediating effect analysis in this study (71). When the sample size was set to the actual number in this study (*N* = 1148) and the variable correlation coefficients were based on the actual findings, the results indicated a power of 1, suggesting that the sample size for the chain mediating analysis in this study is sufficient.

## 4 Discussion

The current study investigated the relationship between childhood maltreatment and aggressive behavior among Chinese college students, examining the mediating roles of authenticity and alexithymia. Our findings indicate that childhood maltreatment can predict aggressive behavior in college students both directly and through a chain-mediated indirect pathway involving authenticity and alexithymia.

Firstly, we found a close association between childhood maltreatment and aggressive behavior among college students. This finding further validates Ecological Systems Theory. As a microenvironmental factor, childhood maltreatment promotes aggressive behavior in individuals, and this negative impact can persist into adulthood. Based on Attachment Theory, this is because individuals who have been abused internalize this negative interpersonal relationship pattern, leading them to more easily experience attachment anxiety and attachment avoidance, which subsequently triggers aggressive behavior. This finding is consistent with previous studies (18, 72). For instance, Chen and colleagues conducted a survey on 809 Chinese college students and found that emotional abuse during childhood can positively predict aggression (73).

Secondly, our study demonstrated that authenticity partially mediates the relationship between childhood maltreatment and aggressive behavior among college students, as hypothesized. On one hand, we confirmed a significant negative correlation between childhood maltreatment and authenticity among college students, consistent with the Self-Determination Theory (28). When individuals’ feelings and behaviors expressed in interactions with caregivers are not acknowledged or supported, it intensifies self-doubt and self-denial, making individuals less inclined to express their true selves. To survive and minimize harm, individuals may present an inauthentic self and comply with behaviors that hinder the development of authenticity (29). On other hand, authenticity showed a negative correlation with aggressive behavior among college students, aligning with the Meaning Maintenance Model. Low authenticity reduces an individual’s self-worth, life meaning, and life satisfaction (74, 75), triggering compensatory behaviors for meaning. Hostility, bias, and aggression have been identified as common compensatory mechanisms (35, 36). Consequently, individuals with lower authenticity are more prone to engaging in aggressive behaviors.

Furthermore, our results indicated that alexithymia partially mediates the relationship between childhood maltreatment and aggressive behavior. Two meta-analysis studies suggest that childhood maltreatment is a risk factor for alexithymia (76, 77). This impact may be achieved through two pathways: physiological (affecting brain structures with emotional function) and psychological (emotional numbing as a self-protecting mechanism) (43, 44, 46). Meanwhile, alexithymia hinders college students from engaging effectively in social interactions, impeding the development of interpersonal relationships. Specifically, due to their deficits in emotional awareness and expression, as well as lower empathy levels (78, 79), they struggle to adopt adaptive strategies (such as calm communication) to resolve problems in stressful interpersonal interactions (32), there-by exacerbating aggressive behaviors.

Lastly and more importantly, this study provides preliminary evidence of a chain-mediating effect of authenticity and alexithymia in the association between child-hood maltreatment and aggressive behavior. Within the framework of the GAM, individuals who experienced childhood maltreatment tend to suppress the expression of their true selves to avoid negative consequences. While this adaptive response may temporarily function, the long-term development of a “false self” (80) under this pattern may hinder the recognition and expression of emotions, leading to alexithymia. Sequent, suppressed emotions and feelings do not dissipate with age; instead, they may sensitize individuals to perceive (perhaps nonexistent) threats, triggering excessive self-protective mechanisms and aggression towards others. These findings further expand the GAM and provide important insights into the occurrence of aggressive behavior among college students.

## 5 Contributions

Our research has the following contributions. Firstly, although there is evidence that childhood maltreatment increases aggressive behavior in college students, the psychological mechanisms underlying this process still need to be further investigated. To our knowledge, almost no studies have yet explored how childhood maltreatment, authenticity, and alexithymia collectively influence aggressive behavior in college students. We explained this mechanism through a chain mediation model, which helps to deepen our understanding of the relationship between childhood maltreatment and aggressive behavior.

Secondly, our research further supports the Ecological Systems Theory. The findings reveal that a negative family environment (childhood maltreatment) can have a detrimental impact on individual development. This impact is not only current and temporary but can also extend into early adulthood.

Finally, our research also further supports the GAM. The GAM emphasizes that environmental factors can influence aggressive behavior through personality factors. Consistent with the assumptions of GAM, our study reveals that abuse from parents or guardians can promote alexithymia by suppressing authenticity, which in turn increases an individual’s aggressive behavior. This deepens our understanding of the formation mechanisms of individual aggressive behavior.

## 6 Implications

This study holds significant educational and societal implications. It highlights the lasting impact of childhood maltreatment on individual development, stressing the importance of early prevention and intervention at both family and community levels. It suggests that public awareness around parenting practices and the potential harm of neglect or harsh treatment should be increased, with a broader emphasis on providing accessible education and training on positive parenting techniques.

Moreover, this research sheds light on factors contributing to aggressive behaviors commonly observed among college students, offering insights for future interventions. For educators, understanding and recognizing the emotional needs of students with a history of maltreatment is crucial in creating a supportive environment that encourages these individuals to express their authentic selves. Special attention and targeted intervention should be directed toward individuals exhibiting alexithymia, aiming to reduce the incidence of aggressive behaviors and promote both mental and physical well-being within the student population.

## 7 Limitations and future direction

This study has several limitations. Firstly, it conceptualizes childhood maltreatment as a unified construct without distinguishing between its different subtypes, which may vary in their impact—such as emotional abuse having potentially stronger effects than physical abuse. Future research could explore and discuss these differences through comparative analyses of different maltreatment types. Secondly, this study relies on cross-sectional data to examine the mediation analysis. While this approach allows us to identify associations between variables, it does not establish causal relationships. Future studies employing longitudinal or cross-lagged designs would be valuable to confirm the directionality of the effects observed in this study and to provide a more robust understanding of the underlying mechanisms. Thirdly, the reliance on self-report methods for participants to recall past maltreatment experiences may be susceptible to memory biases. Future research could integrate multiple assessment methods, such as implicit association tests, to complement self-report data. Lastly, exploring other potential mediating or moderating variables, such as intolerance of uncertainty or self-pity, could provide additional insights.

## 8 Conclusions

1. Childhood Maltreatment positively predicts aggressive behavior among college students.
2. Authenticity mediates the relationship between childhood maltreatment and aggressive behavior.
3. Alexithymia mediates the relationship between childhood maltreatment and aggressive behavior.
4. Authenticity and Alexithymia play the serial mediating role in the relationship between childhood maltreatment and aggressive behavior.

## Data Availability

The original contributions presented in the study are included in the article/supplementary material. Further inquiries can be directed to the corresponding author.
